# Supplementary material for: High Expression of PD-L1 Is Associated with Better Survival in Pancreatic/Periampullary Cancers and Correlates with Epithelial to Mesenchymal Transition
Source: Diagnostics (Basel). 2021 Mar 26;11(4):597. doi: 10.3390/diagnostics11040597 (PMC8065840; doi:10.3390/diagnostics11040597)
Supplement: Supplementary file 1 [file diagnostics-11-00597-s001.pdf]

diagnostics

MDPI

Article

High Expression of PD-L1 is Associated with Better Survival in Pancreatic/Periampullary Cancers and Correlates with Epithelial to Mesenchymal Transition

Supplementary Materials:

Table S1. Result of univariable cox proportional hazards regression analysis using death event as status variable and overall survival time as survival time

Variable	Subgroup	HR (95%CIs)	P value
Age		1.024 (0.992 - 1.056)	0.141
Sex			
	Female vs. Male	1.031 (0.607 - 1.752)	0.909
Location			**< 0.001****
	Pancreatic Head vs. AOV	11.795 (4.649 - 29.925)	**< 0.001****
	Distal CBD vs.AOV	3.609 (1.466 - 8.886)	**0.005****
T7			1
	T1 vs Tis	7364538.688 (0 - Inf)	0.996
	T2 vs. Tis	5053164.568 (0 - Inf)	0.996
	T3 vs. Tis	19062328.45 (0 - Inf)	0.996
	T4 vs. Tis	24298595.997 (0 - Inf)	0.996
T8			1
	T1 vs Tis	7047555.234 (0 - Inf)	0.996
	T2 vs. Tis	8921396.166 (0 - Inf)	0.996
	T3 vs. Tis	13294799.292 (0 - Inf)	0.996
	T4 vs. Tis	18021584.76 (0 - Inf)	0.996
Gross type			**0.013***
	Infiltrative vs. Fungative	4.156 (1.643 - 10.517)	**0.003****
	Ulcerofungative vs. Fungative	0 (0 - Inf)	0.997
	Sessile vs. Fungative	12.437 (2.872 - 53.858)	**< 0.001****
	Solid vs. Fungative	0 (0 - Inf)	0.997
Ulcer			
	Present vs. Absent	0.613 (0.216 - 1.742)	0.358
Size		1.336 (1.125 - 1.587)	**< 0.001****
Size_4.5 cm			
	≥4.5 cm vs. <4.5 cm	1.876 (1.056 - 3.332)	**0.032***
Radial resection margin			
	Present vs. Absent	2.177 (0.85 - 5.571)	0.105
N1		1.425 (1.25 - 1.626)	**< 0.001****

N2		1.001 (0.965 - 1.037)	0.967
N stage			
	N1 vs. N0	4.005 (2.304 - 6.961)	**< 0.001****
M stage			
	M1 vs. M0	4.142 (1.919 - 8.936)	**< 0.001****
Lymphatic invasion			
	Present vs. Absent	3.915 (2.243 - 6.836)	**< 0.001****
Perineural invasion			
	Present vs. Absent	2.915 (1.62 - 5.246)	**< 0.001****
Vascular invasion			
	Present vs. Absent	3.088 (1.419 - 6.718)	**0.004****
Histologic grade			**0.01****
	Moderate vs. Well	2.603 (1.327 - 5.104)	**0.005****
	Poorly vs. Well	4.601 (1.245 - 17.004)	**0.022***
Fibrosis			1
	Mild vs. None	11804768.899 (0 - Inf)	0.996
	Moderate vs. None	28264925.781 (0 - Inf)	0.996
	Severe vs. None	56880557.21 (0 - Inf)	0.996
Degree of Inflammation			0.072
	Moderate vs. Mild	0.619 (0.347 - 1.106)	0.105
	Severe vs. Mild	0.311 (0.105 - 0.927)	**0.036***
Histologic subtype			**0.004****
	Prone to Pancreaticobiliary subtype vs. Pancreaticobiliary subtype	0.519 (0.291 - 0.926)	**0.026***
	Prone to Intestinal subtype vs. Pancreaticobiliary subtype	0.287 (0.108 - 0.762)	**0.012***
	Intestinal vs. Pancreaticobiliary subtype	0.174 (0.052 - 0.583)	**0.005****
CK7			0.177
	1+ vs. 0	2.303 (0.383 - 13.838)	0.362
	2+ vs. 0	3.474 (0.797 - 15.139)	0.097
	3 +vs. 0	4.298 (1.031 - 17.914)	**0.045***
CK20			**0.039***
	1+ vs. 0	0.523 (0.244 - 1.122)	0.096
	2+ vs. 0	0.379 (0.149 - 0.965)	**0.042***
	3+ vs. 0	0.335 (0.103 - 1.088)	0.069
CDX2			**0.002****
	1+ vs. 0	0.64 (0.35 - 1.169)	0.147
	2+ vs. 0	0.353 (0.157 - 0.794)	**0.012***
	3+vs. 0	0.186 (0.07 - 0.489)	**< 0.001****
IGF1			0.439

		1+ vs. 0	1.098 (0.43 - 2.809)	0.845
		2 +vs. 0	0.645 (0.222 - 1.88)	0.422
		3+ vs. 0	0.686 (0.181 - 2.599)	0.579
FGFR				**0.003****
		2+ vs. 1+	0.268 (0.089 - 0.808)	**0.019***
		3+ vs. 1+	0.154 (0.05 - 0.473)	**0.001****
VEGF				**0.037***
		2+vs. 1+	0.407 (0.198 - 0.837)	**0.015***
		3+ vs. 1+	0.385 (0.166 - 0.894)	**0.026***
PD1	Score			0.734
		1+vs. 0	0.998 (0.426 - 2.34)	0.997
		2+ vs. 0	0.563 (0.134 - 2.359)	0.432
	Score			0.648
		(1+ or 2+) vs. 0	0.839 (0.395 - 1.781)	0.648
PDL1	Score1			0.118
		2+ vs. 1+	0.759 (0.328 - 1.757)	0.519
		3+ vs. 1+	0.469 (0.2 - 1.101)	0.082
	Score1			0.052
		3+ vs. (1+ or 2+)	0.586 (0.342 - 1.005)	0.052
	Score2			**0.012***
		2+ vs. 1+	0.925 (0.481 - 1.776)	0.814
		3+ vs. 1+	0.4 (0.199 - 0.805)	**0.010***
	Score2			
		3+ vs. (1+ or 2+)	0.42 (0.237 - 0.745)	**0.003****
PDL2	Score1			0.450
		1+ vs. 0	0.688 (0.133 - 3.563)	0.656
		2+ vs. 0	1.116 (0.258 - 4.827)	0.883
		3+ vs. 0	0.701 (0.167 - 2.945)	0.628
	Score1			0.292
		2+ vs. (0 or 1+)	1.478 (0.617 - 3.541)	0.381
		3+ vs. (0 or 1+)	0.928 (0.407 - 2.118)	0.859
	Score1			0.214
		3+ vs. (0 or 1+ or 2+)	0.712 (0.417 - 1.216)	0.214
	Score1			0.846
		(2+ or 3+) vs. (0 or 1+)	1.082 (0.489 - 2.395)	0.846
	Score2			0.262
		1+ vs. 0	1.497 (0.599 - 3.738)	0.388
		2+ vs. 0	1.391 (0.556 - 3.478)	0.481
		3+ vs. 0	0.728 (0.261 - 2.033)	0.545
	Score2			0.643
		(1+ or 2+ or 3+) vs. 0	1.223 (0.522 - 2.87)	0.643

Score2			0.140
	(1+ or 2+) vs. 0	1.442 (0.609 - 3.414)	0.405
	3+ vs. 0	0.728 (0.261 - 2.032)	0.544

HR: hazard ratio, Significant values ($P < 0.05$) have been marked with bold.

Table S2. Result of multivariable cox proportional hazards regression analysis using death event as response and overall survival time as the time variable.

Variable(response=Death Event)	Subgroup	HR (95%CIs)	P-value
Location			**< 0.001****
	Pancreatic head vs. AOV	7.355 (2.713 - 19.943)	**< 0.001****
	Distal CBD vs. AOV	2.855 (1.115 - 7.313)	**0.029***
N stage			**0.001****
	N1 vs. N0	2.887 (1.531 - 5.445)	**0.001****
M stage			**0.004****
	M1 vs. M0	3.488 (1.479 - 8.226)	**0.004****
FGFR			**0.004****
	2+ vs. 1+	0.191 (0.06 - 0.611)	**0.005****
	3+ vs. 1+	0.128 (0.038 - 0.428)	**< 0.001****
PDL1 Score2			
	3+ vs. (1+ or 2+)	0.48 (0.259 - 0.887)	**0.019***

HR: hazard ratio, Significant values ($P < 0.05$) have been marked with bold.

Table S3. Result of univariable cox proportional hazards regression analysis using recurrence event as status variable and Disease-free survival time as survival time.

Variable	Subgroup	HR (95%CIs)	P-value
Age		1.034 (1.001 - 1.069)	**0.042***
Sex			
	Female vs. Male	0.964 (0.554 - 1.679)	0.898
Location			**0.008****
	Pancreatic head vs. AOV	3.372 (1.556 - 7.31)	**0.002****
	Distal CBD vs. AOV	1.82 (0.882 - 3.758)	0.105
T7			1
	T1 vs. Tis	6267620.358 (0 - Inf)	0.996
	T2 vs. Tis	7587718.783 (0 - Inf)	0.996
	T3 vs. Tis	14571449.458 (0 - Inf)	0.996
	T4 vs. Tis	10132566.792 (0 - Inf)	0.996
T8			1

	T1 vs. Tis	6623316.928 (0 - Inf)	0.996
	T2 vs. Tis	9328025.227 (0 - Inf)	0.996
	T3 vs. Tis	13466073.961 (0 - Inf)	0.996
	T4 vs. Tis	11480091.662 (0 - Inf)	0.996
Gross type			0.994
	Infiltrative vs. Fungative	2.157 (1.003 - 4.639)	**0.049***
	Ulcerofungative vs. Fungative	1.046 (0.13 - 8.383)	0.966
	Sessile vs. Fungative	1.52 (0.187 - 12.343)	0.695
	Solid vs. Fungative	0 (0 - Inf)	0.996
Ulcer			
	Present vs. Absent	0.976 (0.378 - 2.52)	0.961
Size		1.265 (1.06 - 1.509)	**0.009****
Size_4.5 cm			
	≥4.5 cm vs. <4.5 cm	1.836 (1.004 - 3.357)	**0.049***
Radial resection margin			
	Present vs. Absent	1.896 (0.678 - 5.301)	0.223
N1		1.278 (1.111 - 1.471)	**< 0.001****
N2		1.004 (0.967 - 1.042)	0.848
N stage			
	N1 vs. N0	2.563 (1.455 - 4.516)	**0.001****
M stage			
	M1 vs. M0	0.314 (0.043 - 2.283)	0.253
Lymphatic invasion			
	Present vs. Absent	2.998 (1.701 - 5.284)	**< 0.001****
Perineural invasion			
	Present vs. Absent	1.727 (0.981 - 3.041)	0.058
Vascular invasion			
	Present vs. Absent	1.726 (0.728 - 4.092)	0.215
Histologic grade			**0.003****
	Moderate vs. Well	1.967 (1.016 - 3.809)	**0.045***
	Poorly vs. Well	7.425 (2.287 - 24.107)	**< 0.001****
Degree of Fibrosis			1
	Mild vs. None	23201558.334 (0 - Inf)	0.996
	Moderate vs. None	23674351.857 (0 - Inf)	0.996
	Severe vs. None	58119540.116 (0 - Inf)	0.996
Degree of inflammation			0.807
	Moderate vs. Mild	0.892 (0.474 - 1.678)	0.722
	Severe vs. Mild	0.726 (0.277 - 1.9)	0.514
Histologic subtype			0.109
	Prone topancreaticobiliary vs. Pancreaticobiliary subtype	0.666 (0.358 - 1.242)	0.201

		Prone to intestinal subtype vs. Pancreaticobiliary subtype	0.456 (0.178 - 1.167)	0.102
		Intestinal subtype vs. Pancreaticobiliary subtype	0.299 (0.1 - 0.893)	**0.031***
CK7				0.387
		1+ vs. 0	2.614 (0.477 - 14.326)	0.268
		2+ vs. 0	3.346 (0.768 - 14.569)	0.108
		3+ vs. 0	3.426 (0.817 - 14.375)	0.092
CK20				0.395
		1+ vs. 0	0.533 (0.235 - 1.21)	0.133
		2+ vs. 0	0.683 (0.3 - 1.554)	0.363
		3+ vs. 0	0.664 (0.257 - 1.713)	0.397
CDX2				**0.035***
		1+ vs. 0	1.033 (0.544 - 1.961)	0.921
		2+ vs. 0	0.534 (0.228 - 1.256)	0.151
		3+ vs. 0	0.313 (0.122 - 0.799)	**0.015***
IGF1				0.256
		1+ vs. 0	0.938 (0.393 - 2.241)	0.886
		2+ vs. 0	0.48 (0.169 - 1.362)	0.168
		3+ vs. 0	0.488 (0.12 - 1.989)	0.317
FGFR				**0.01***
		2+ vs. 1+	0.445 (0.151 - 1.314)	0.143
		3+ vs. 1+	0.227 (0.075 - 0.689)	**0.009****
VEGF				0.199
		2+ vs. 1+	0.536 (0.252 - 1.139)	0.105
		3+ vs. 1+	0.473 (0.196 - 1.142)	0.096
PD1	Score			0.549
		1+ vs. 0	0.623 (0.224 - 1.732)	0.364
		2+ vs. 0	0.616 (0.147 - 2.586)	0.508
	Score			
		(1+ or 2+) vs. 0	0.621 (0.264 - 1.457)	0.273
PDL1	Score1			0.303
		2+ vs. 1+	0.88 (0.336 - 2.307)	0.795
		3+ vs. 1+	0.583 (0.219 - 1.551)	0.280
	Score1			
		3+ vs. (1+ or 2+)	0.649 (0.372 - 1.133)	0.129
	Score2			**0.032***
		2+ vs. 1+	0.847 (0.43 - 1.669)	0.632
		3+ vs. +1	0.417 (0.203 - 0.855)	**0.017***
	Score2			
		3+ vs. (1+ or 2+)	0.462 (0.256 - 0.833)	**0.010***
PDL2	Score1			0.336

	1+ vs. 0	0.506 (0.126 - 2.03)	0.336
	2+ vs. 0	0.685 (0.2 - 2.344)	0.546
	3+ vs. 0	0.429 (0.129 - 1.428)	0.168
Score1			0.310
	2+ vs. (0 or 1+)	1.131 (0.503 - 2.539)	0.766
	3+ vs. (0 or 1+)	0.709 (0.331 - 1.521)	0.377
Score1			
	3+ vs. (0 or 1+ or 2+)	0.656 (0.378 - 1.14)	0.135
Score1			
	(2+ or 3+) vs. (0 or 1+)	0.837 (0.407 - 1.719)	0.627
Score2			0.181
	1+ vs. 0	1.259 (0.55 - 2.884)	0.585
	2+ vs. 0	1.168 (0.506 - 2.698)	0.716
	3+ vs. 0	0.487 (0.174 - 1.362)	0.171
Score2			
	(1+ or 2+ or 3+) vs. 0	0.996 (0.466 - 2.126)	0.991
Score2			0.090
	(1+ or 2+) vs. 0	1.214 (0.563 - 2.614)	0.621
	3+ vs. 0	0.487 (0.174 - 1.362)	0.170

HR: hazard ratio, Significant values (*P* < 0.05) have been marked with bold.

Table S4. Result of multivariable cox proportional hazards regression analysis using recurrence event as response and Disease-free survival time as the time variable**.**

Variable	Subgroup	HR (95%CIs)	P-value
Age		1.046 (1.011 - 1.083)	**0.01***
Lymphatic invasion			
	Present vs. Absen	3.289 (1.838 - 5.884)	**< 0.001****
PDL1 Score2			
	3+ vs. (1+ or 2+)	0.474 (0.262 - 0.86)	**0.014***

HR: hazard ratio, Significant values (*P* < 0.05) have been marked with bold.

Figure S1.

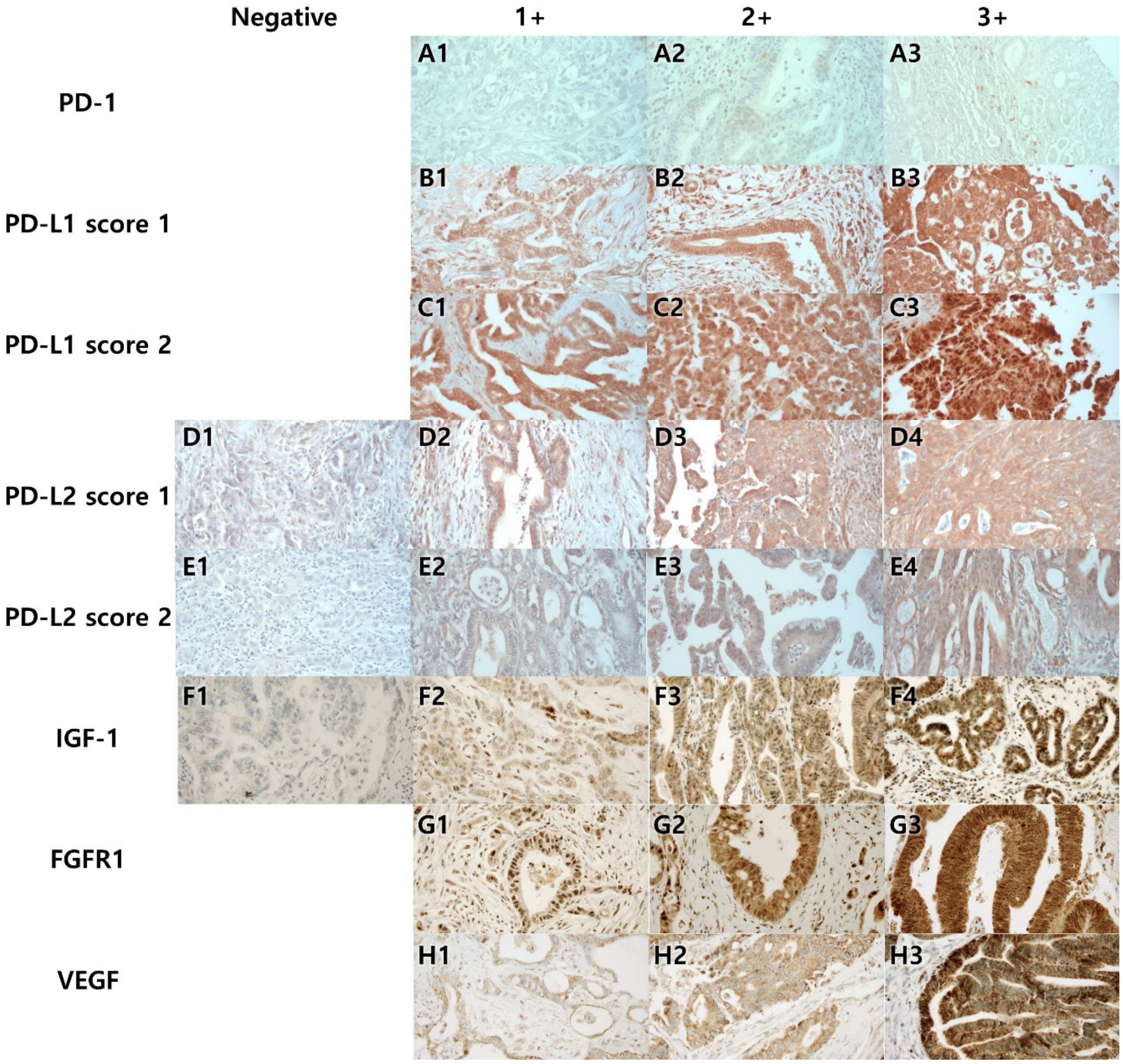

Figure S1. Representative images of the immunohistochemical stainings using the tissue microarray of periampullary/ periampullary cancers (x400). (A) PD-1, (B) PD-L1 score 1 and (C) score 2, (D) PD-L2 score 1 and (E) score 2, (F) IGF-1 (G) FGFR1, and (H) VEGF, with negative (no expression), 1+ (low expression), 2+ (moderate expression) and 3+ (high expression) intensity.

Figure S2

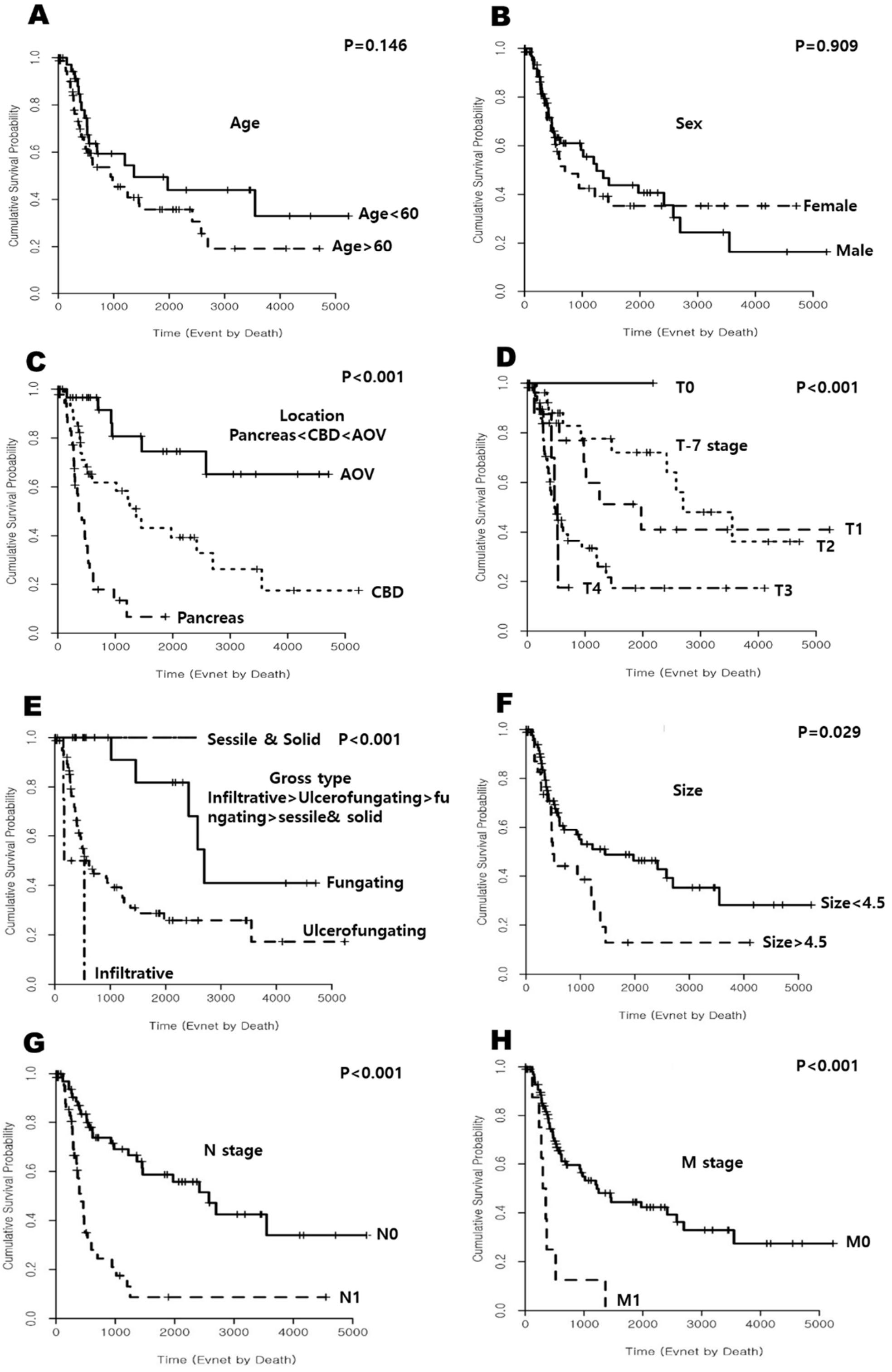

Figure S2. Kaplan-Meier plots on the relationship between overall survival and clinical parameters in periampullary/pancreatic cancer patients. No significant relationship found according to (A) age and (B) sex, whereas there was significant difference according to (C) location, (D) T stage, (E) gross type, (F) size, (G) N stage, and (H) M stage

Figure S3.

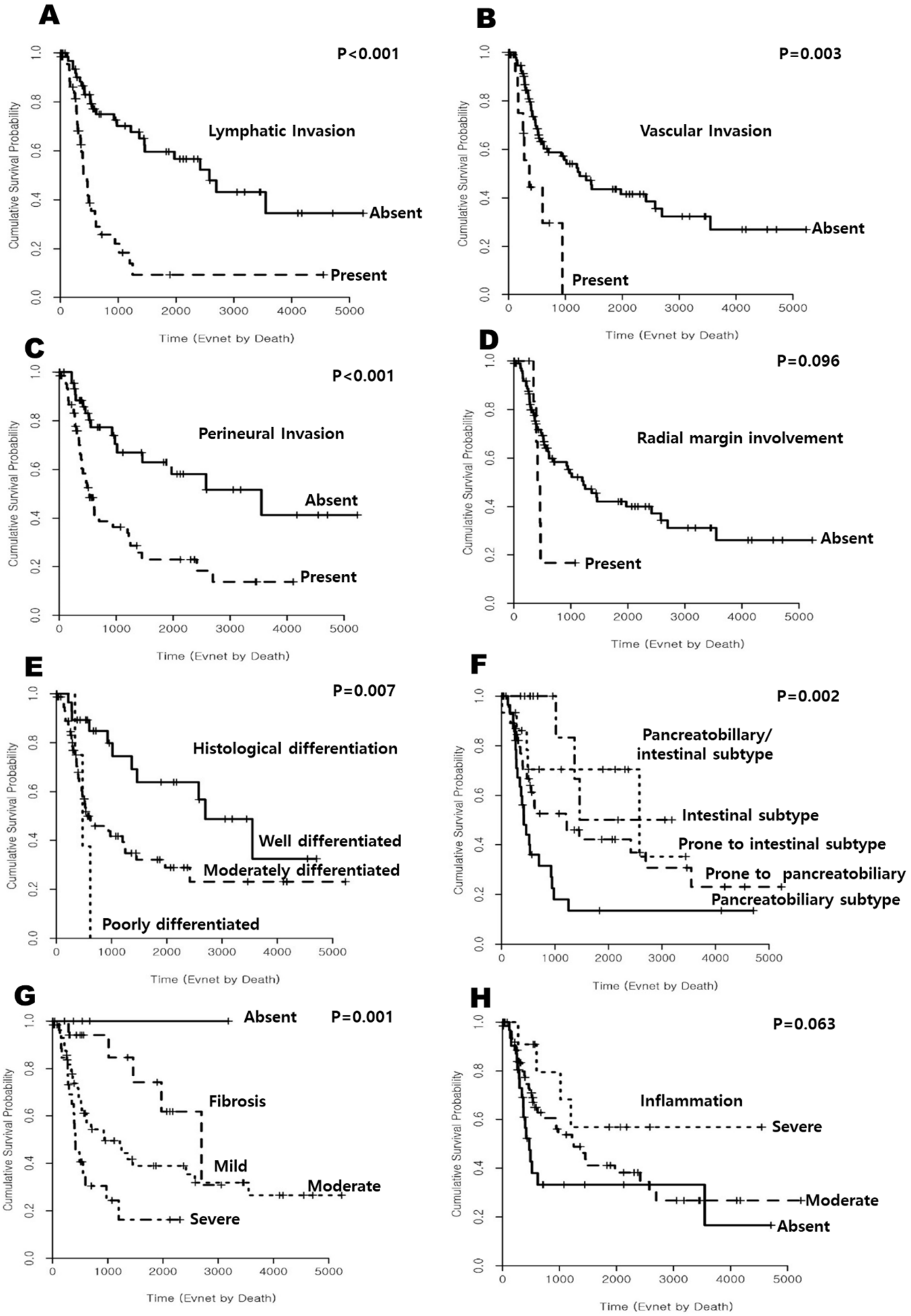

Figure S3. Kaplan-Meier plots on the relationship between overall survival and the pathological parameters in the periampullary/pancreatic cancers patients. There was significant relationship according to (A) lymphatic invasion, (B) vascular Invasion, (C) perineural invasion, (E) histological differentiation, (F) pancreatobillary/intestinal type, and (G) fibrosis, while there was no significant difference according to (D) radial margin involvement, and (H) inflammation.

Figure S4.

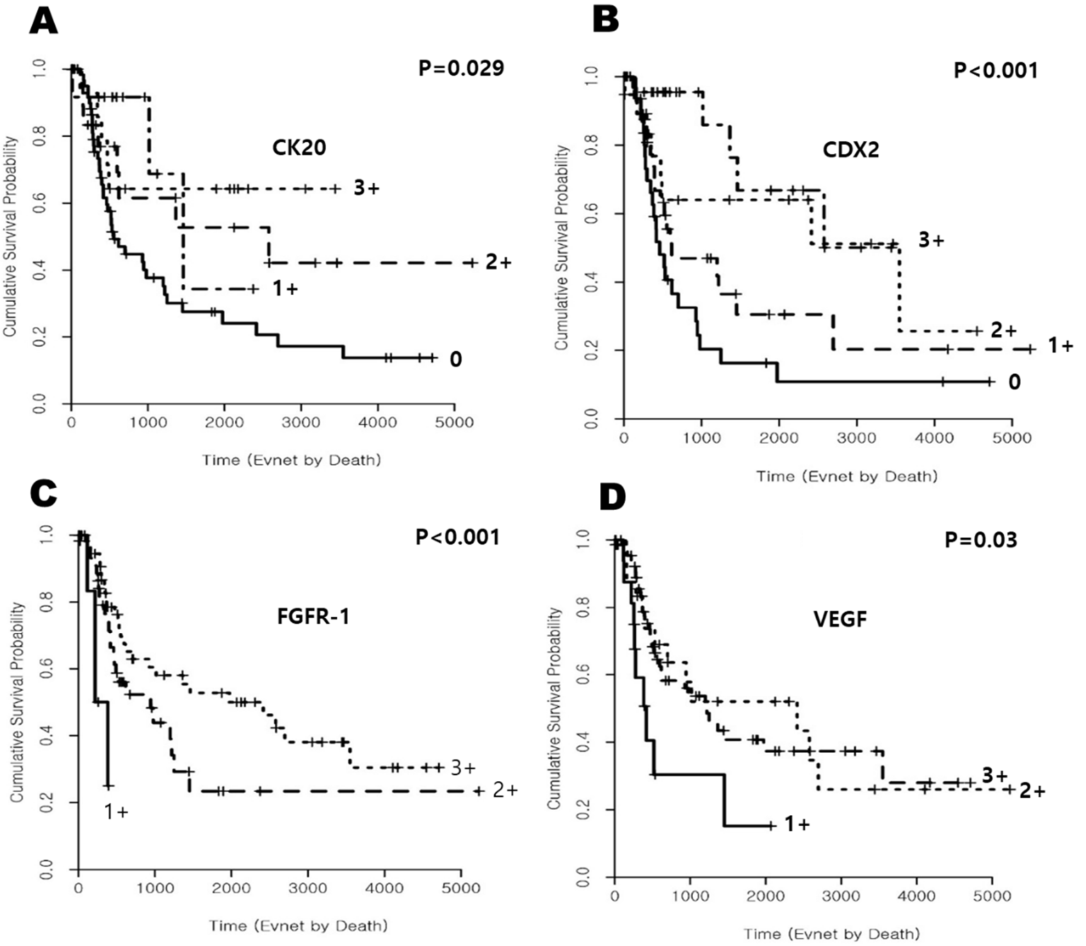

Figure S4. Kaplan-Meier plots survival analysis on the relationship between overall survival and expression level of the IHC markers in periampullary/pancreatic cancers patients. There was significant relationship according to (A) CK20, (B) CDX2, (C) FGFR1, and (D) VEGF.

Figure S5.

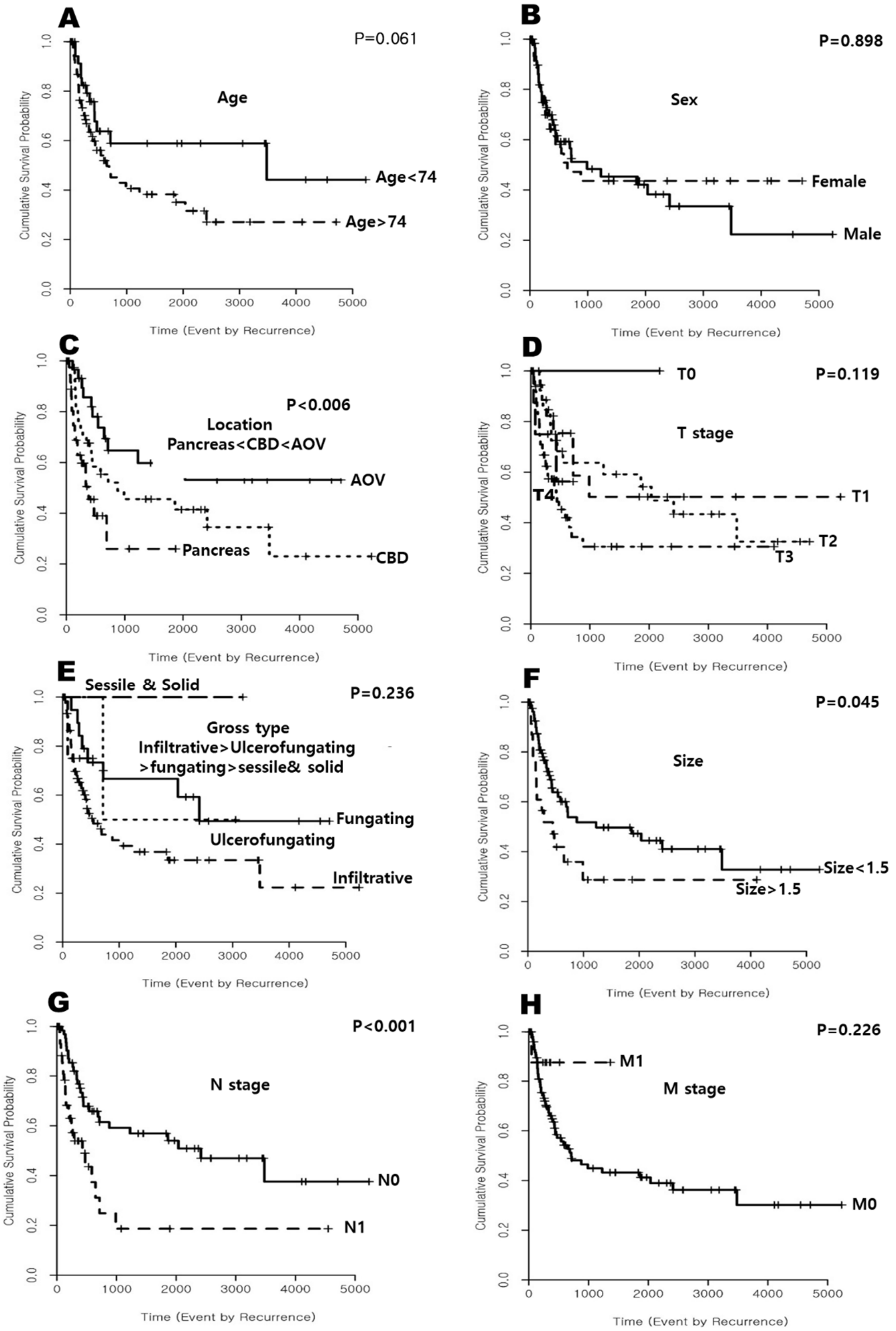

Figure S5. Kaplan-Meier plots on the relationship between disease free survival and clinical parameter in recurrent Periampullary/Pancreatic cancers patients. There was significant no relationship according to (A) age, (B) sex, (E) gross type, and M stage. While there was significant relationship according to, (C) location, (D) T stage, (F) Size, and (G) N stage.

Figure S6

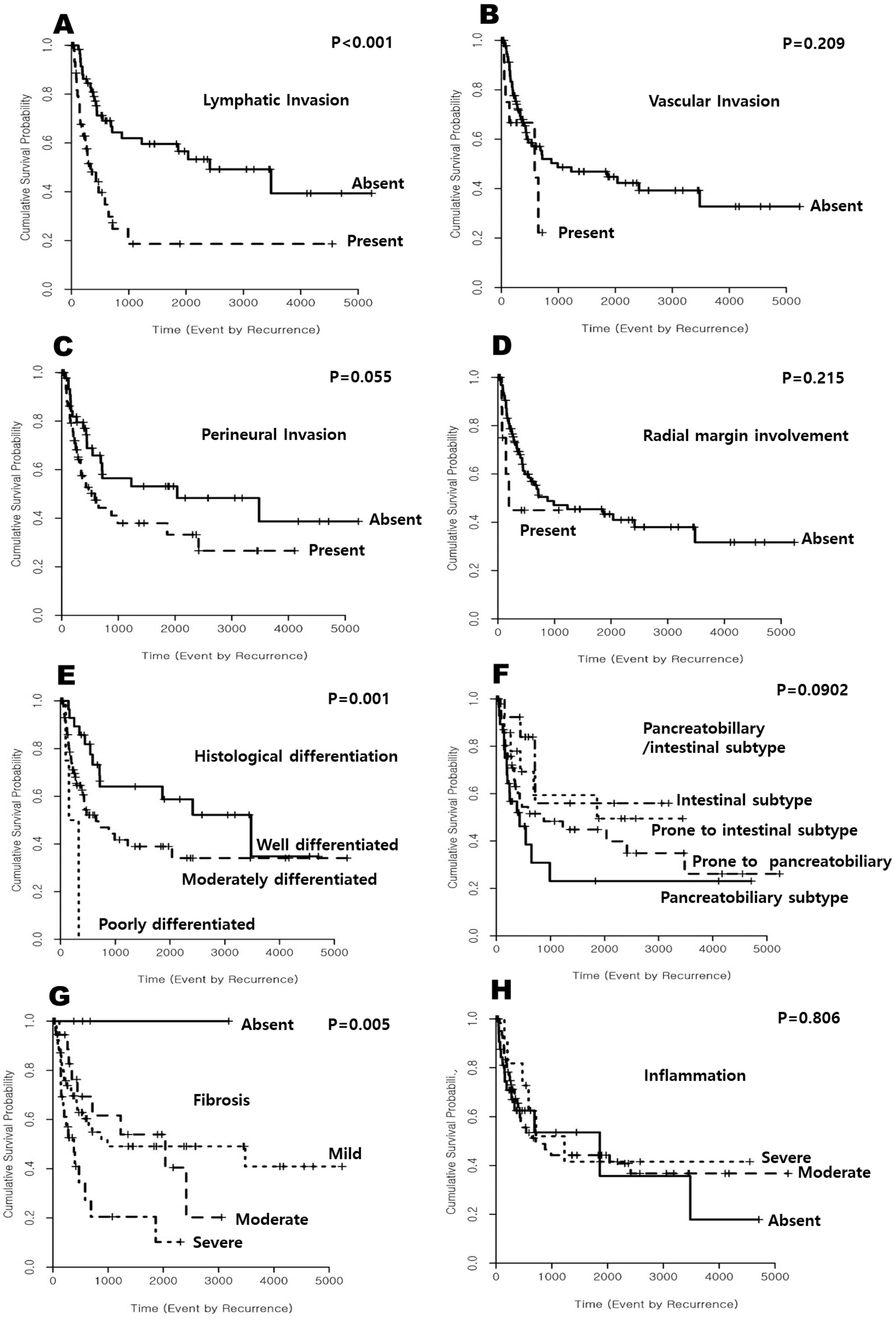

Figure S6. Kaplan-Meier plots on the relationship between disease free survival and pathological parameter in recurrent Periampullary/Pancreatic cancers patients. There was significant relationship according to (A) lymphatic invasion, (E) histological differentiation, and (G) fibrosis, whereas there was no significant difference according to (B) vascular Invasion, (C) perineural invasion, (D) radial margin involvement, (F) pancreatobillary type vs intestinal type, and (H) inflammation.

Figure S7.

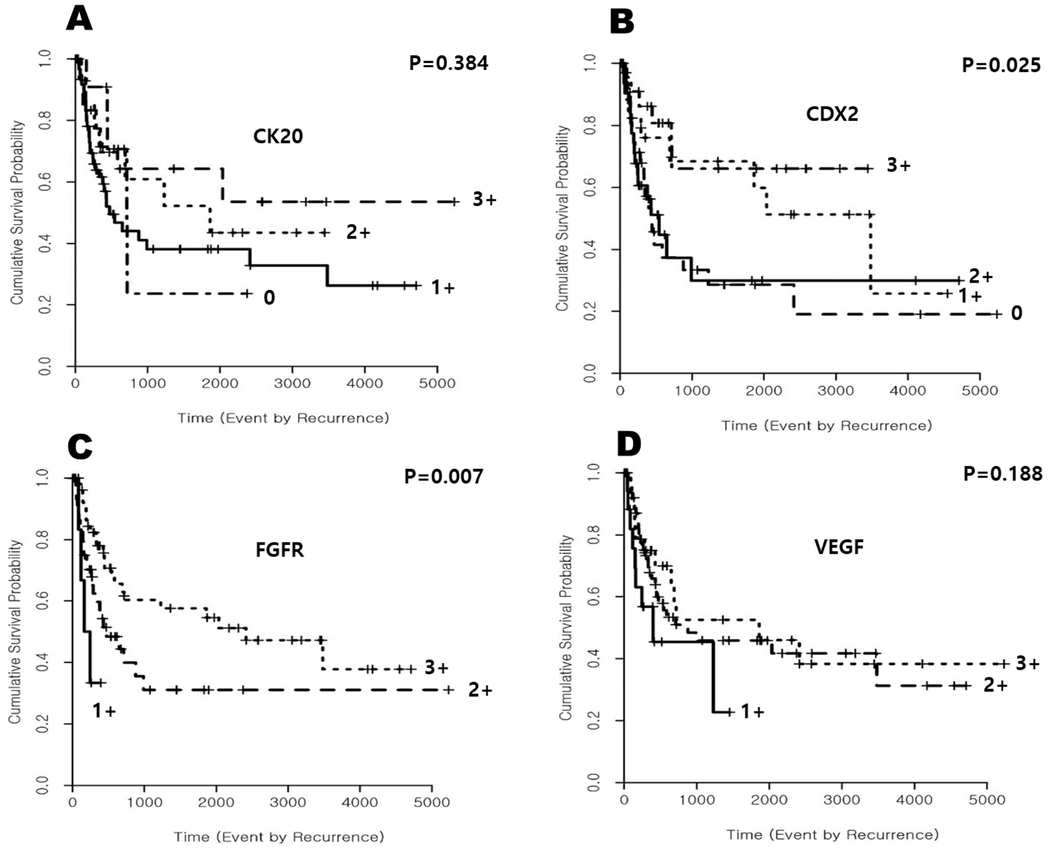

Figure S7. Kaplan-Meier plots on the relationship between disease free survival analysis and IHC markers expression level in recurrent Periampullary/Pancreatic cancers patients. There was significant relationship according to (A) CK20, (B) CDX2, (C) FGFR1, and whereas there was not significant difference according to (D) VEGF.
